# Supplementary material for: Transformation of Tn7 insertion elements across strains of Vibrio fischeri
Source: PLoS One. 2025 Dec 30;20(12):e0338632. doi: 10.1371/journal.pone.0338632 (PMC12752967; doi:10.1371/journal.pone.0338632)
Supplement: S1 File — (PDF) [file pone.0338632.s001.pdf]

# Transformation of Tn*7* insertion elements across strains of *Vibrio fischeri*

RESERVED DOI:
10.17504/protocols.io.kqdg31k11l25/v1

Andrew G. Cecere[1,2], Chris A. Muriel-Mundo[1,2], Derek J. Fisher[3,4], Tim I. Miyashiro[1,2,5]

[1]Department of Biochemistry and Molecular Biology, Pennsylvania State University, University Park, PA, USA;
[2]The One Health Microbiome Center, Huck Institutes of the Life Sciences, Pennsylvania State University, University Park, PA, USA;
[3]Multidisciplinary Biomedical & Biological Sciences, Southern Illinois University Carbondale, Carbondale, IL, USA;
[4]School of Biological Sciences, Southern Illinois University Carbondale, Carbondale, IL, USA;
[5]Correspondence: tim14@psu.edu

Tim I Miyashiro
Pennsylvania State University

**Protocol Info:** Andrew G. Cecere, Chris A. Muriel-Mundo, Derek J. Fisher, Tim I. Miyashiro . Transformation of Tn7 insertion elements across strains of Vibrio fischeri. **protocols.io** **https://protocols.io/view/transformation-of-tn7-insertion-elements-across-st-g9pqbz5mx**

**Created:** September 08, 2025

**Last Modified:** November 04, 2025

**Protocol Integer ID:** 226768

**Funders Acknowledgements:**
**National Institute of General Medical Sciences**
Grant ID: R35 GM152259

## Disclaimer

DISCLAIMER – FOR INFORMATIONAL PURPOSES ONLY; USE AT YOUR OWN RISK
The protocol content here is for informational purposes only and does not constitute legal, medical, clinical, or safety advice, or otherwise; content added to **protocols.io** is not peer reviewed and may not have undergone a formal approval of any kind. Information presented in this protocol should not substitute for independent professional judgment, advice, diagnosis, or treatment. Any action you take or refrain from taking using or relying upon the information presented here is strictly at your own risk. You agree that neither the Company nor any of the authors, contributors, administrators, or anyone else associated with **protocols.io**, can be held responsible for your use of the information contained in or linked to this protocol or any of our Sites/Apps and Services.

## Abstract

This protocol details how to use pLosTfoX-dependent natural transformation to transform non-canonical strains of *Vibrio fischeri* with genetic content at the Tn*7* insertion of strain ES114.

## Materials

Materials needed:
1. Culture tubes, e.g., 14-mL Round-Bottom Polystyrene Test Tubes (Falcon)
2. Defined minimal medium (DMM) with N-acetylglucosamine (GlcNAc) – [50 mM MgSO4, 10 mM CaCl2, 300 mM NaCl, 10 mM KCl, 0.01 mM FeSO4, 0.33 mM K2HPO4, 50 mM Tris-HCl (pH 7.5), 0.2% GlcNAc]. Store at 4°C, use within 24 hours.
3. Chloramphenicol (cam)
4. 125-mL baffled Erlenmeyer flask
5. LBS medium [1% (w/v) tryptone, 0.5% (w/v) yeast extract, 2% (w/v) NaCl, 50 mM Tris–HCl (pH 7.5)], with 1.5% w/v agar for solid medium
6. Shaking incubator at 28°C (New Brunswick)
7. Spectrophotometer and cuvettes (Eppendorf)
8. Genomic DNA (extracted according to manufacturer's instructions, Biosearch Technologies Inc.)
9. LBS + 5.0 µg/mL erythromycin (erm) plates

## Troubleshooting

## Preparation of *Vibrio fischeri* Cultures

1 Initiate a starter culture by inoculating a Falcon tube containing 2 mL DMM-GlcNAc + 2.5 µg/mL cam with an isolated colony of a *V. fischeri* strain harboring pLosTfoX. Incubate starter culture overnight (~16 h) at 28°C shaking at 200 rpm.

0.2 Transfer 20 mL DMM + 2.5 µg/mL cam into a 125-mL baffled Erlenmeyer flask and incubate at 28°C to prewarm media.

0.3 Measure the $OD_{600}$ of the starter culture. (Note: typically $OD_{600}$ = 1.0–1.5)

0.4 Inoculate flask with 1 mL starter culture. Incubate at 28°C shaking at 200 rpm.

## Preparation of natural transformation reactions

0.5 When the turbidity of culture is $OD_{600}$ = 0.25, transfer 0.5 mL of the culture to two Falcon tubes. To one tube (experimental), add 0.5 µg genomic DNA substrate. To the other tube (no DNA control), add equivalent volume of vehicle. Gently vortex both tubes and incubate at room temperature statically overnight.

## Selection of transformants

0.6 Add 0.5 mL LBS to each culture tube. Incubate shaking at 28°C.

0.7 After 90 minutes, plate 0.2 mL onto LBS-erm. To assess total viable cell count, perform 10-fold serial dilutions to $10^{-7}$ using 10-µL volumes into 90 µL LBS. Spot 10-µL volumes of $10^{-2}$–$10^{-7}$ onto LBS plate with pre-drawn grids. Incubate plates at 28°C overnight.

0.8 For each sample, calculate the concentration of viable cells and transformants per mL. Calculate the transformation efficiency by dividing concentration of transformants by the concentration of DNA.

0.9 Streak purify ermR colonies onto LBS-erm for further isolation.
