# Supplementary figures and images for: Transformation of Tn7 insertion elements across strains of Vibrio fischeri
Source: PLoS One. 2025 Dec 30;20(12):e0338632. doi: 10.1371/journal.pone.0338632 (PMC12752967; doi:10.1371/journal.pone.0338632)

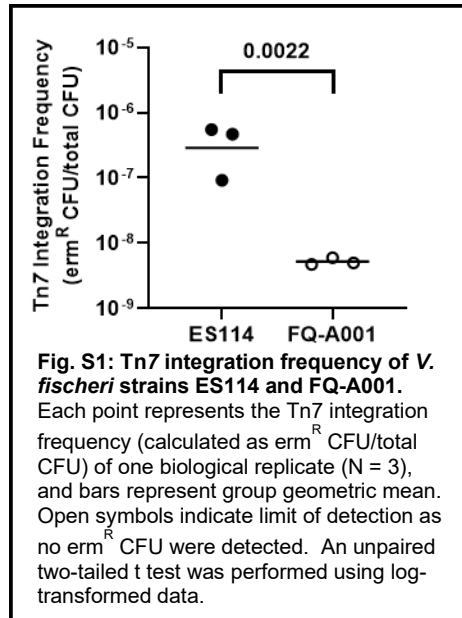

Supplement: S1 Fig — (PDF) [file pone.0338632.s002.pdf]

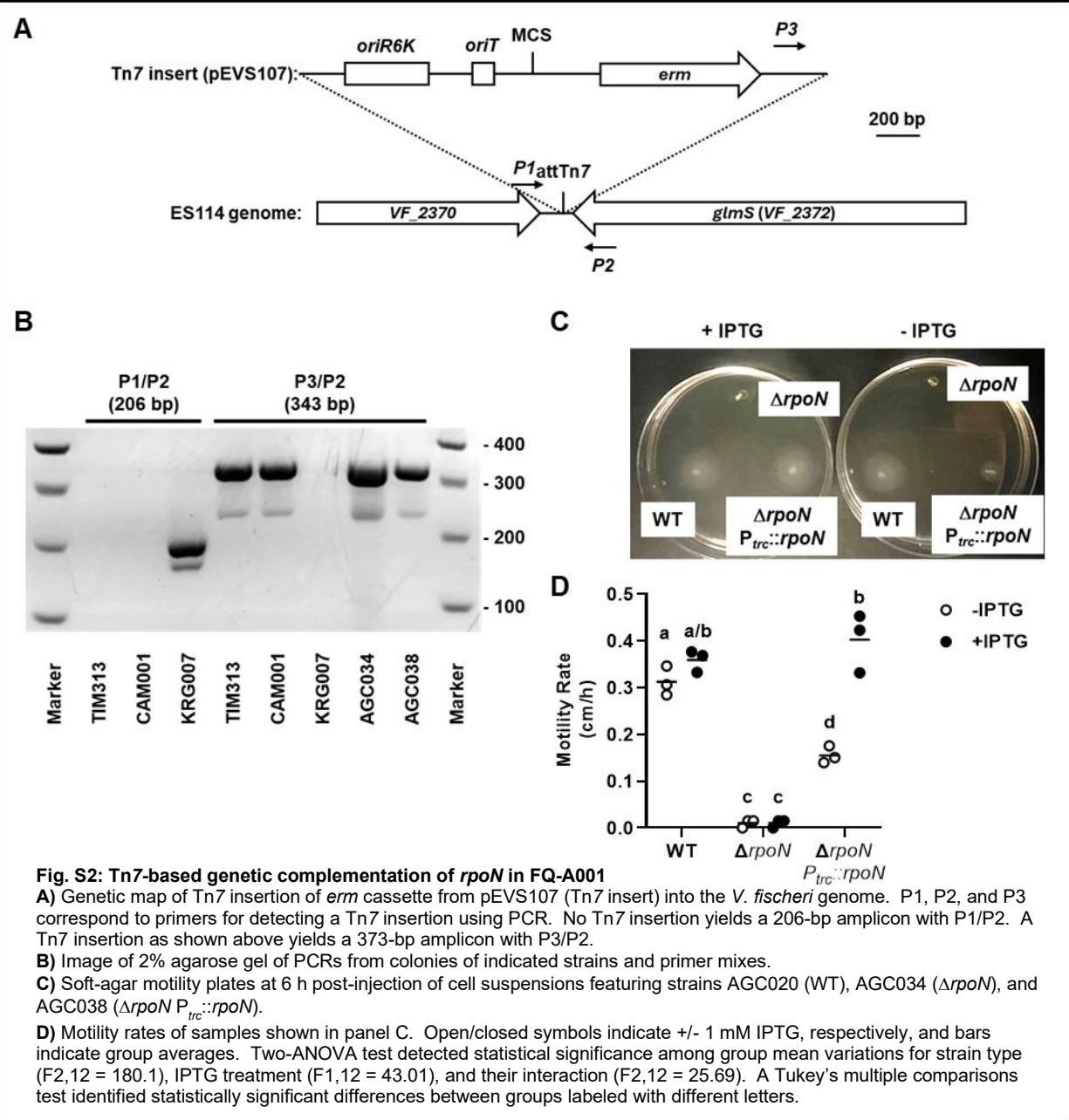

Supplement: S2 Fig — (PDF) [file pone.0338632.s003.pdf]

Raw Image of Fig. S2B

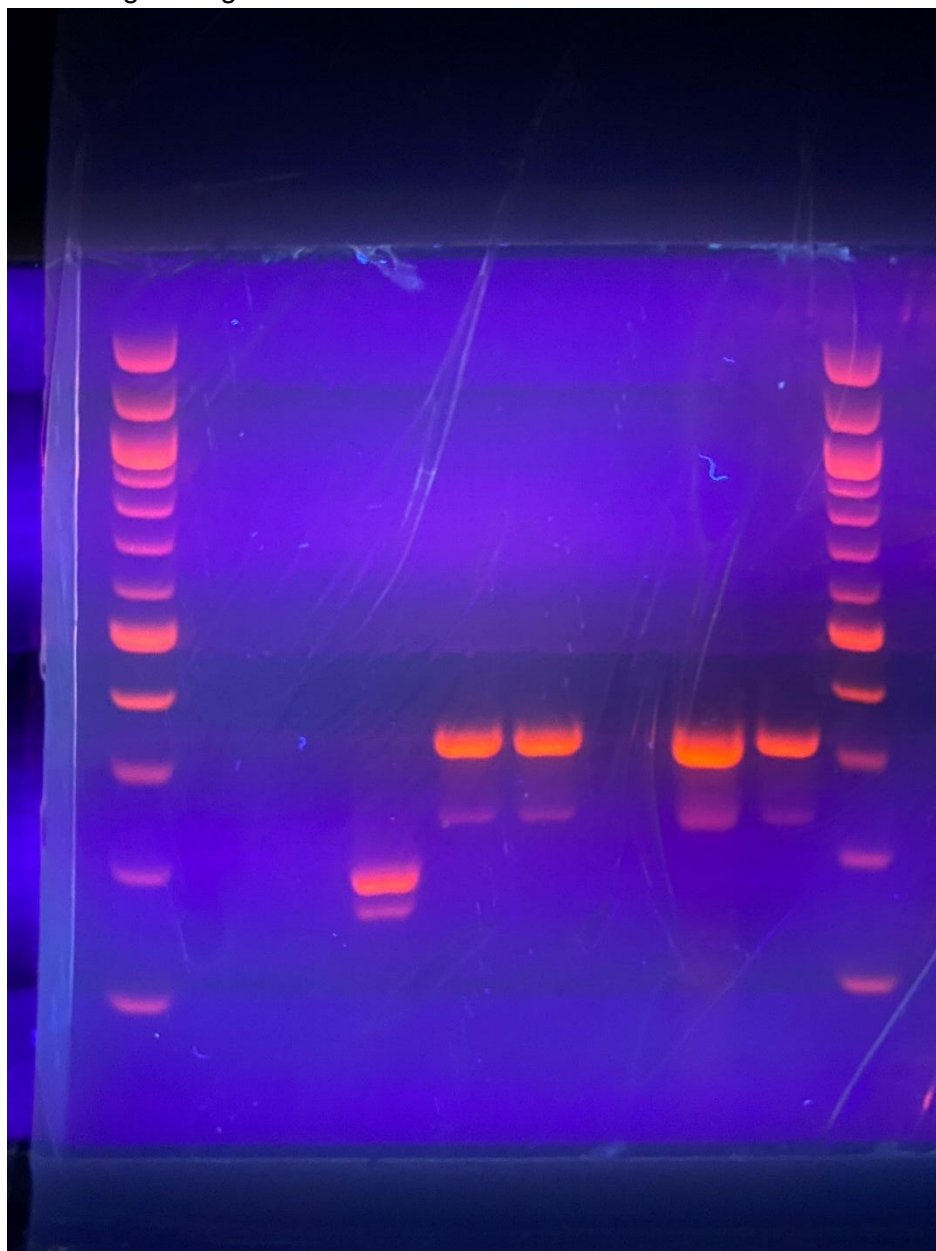

Supplement: S3 Fig — (PDF) [file pone.0338632.s004.pdf]
