# Supplementary material for: Transformation of Tn7 insertion elements across strains of Vibrio fischeri
Source: PLoS One. 2025 Dec 30;20(12):e0338632. doi: 10.1371/journal.pone.0338632 (PMC12752967; doi:10.1371/journal.pone.0338632)
Supplement: Attachment — Submitted filename: 2025 PLoS ONE Lab Protocols-Response to Reviewers.pdf [file pone.0338632.s006.pdf]

Response to Reviewers

PONE-D-25-51250
Transformation of Tn7 insertion elements across strains of Vibrio fischeri
PLOS ONE

Dear Dr. Miyashiro,

Thank you for submitting your manuscript to PLOS ONE. After careful consideration, we feel that it has merit but does not fully meet PLOS ONE's publication criteria as it currently stands. Therefore, we invite you to submit a revised version of the manuscript that addresses the points raised during the review process.

Both the expert reviewer active in the field and myself are enthusiastic about this work. Nonetheless, I would like you to ask you to consider the following minor adjustments to increase accessibility of your work to a broader audience:

– Brief explanation of the rpoN gene in the introduction for context.

Added as requested.

– Demonstration of lack of receptiveness of strain FQ-A001 to attTn7 Tn7 insertion – this could tremendously help colleagues in the field facing similar challenges with diverse marine strains.

This data was included in the original manuscript as Fig. S1, where we show data comparing Tn*7* integration frequency between ES114 (type strain) and FQ-A001. FQ-A001 was at the limit of detection (no Tn*7* mutants were obtained).

– Include details on selection against donor cells.

No counterselection was necessary because the higher-salt medium and lower incubation temperature (28°C) promote the growth of *V. fischeri* over *E. coli*. This detail is now stated in the manuscript.

– Include details on the context of the motility in the context of successful transformation.

Introduction now provides requested context for motility.

– A gentle restructuring of results (e.g., introduction of sub-headers).

Sub-headers have been added as requested.

Please submit your revised manuscript by Dec 18 2025 11:59PM. If you will need more time than this to complete your revisions, please reply to this message or contact the journal office at plosone@plos.org. When you're ready to submit your revision, log on to https://www.editorialmanager.com/pone/ and select the 'Submissions Needing Revision' folder to locate your manuscript file.

Please include the following items when submitting your revised manuscript:

- A rebuttal letter that responds to each point raised by the academic editor and reviewer(s). You should upload this letter as a separate file labeled 'Response to Reviewers'.
- A marked-up copy of your manuscript that highlights changes made to the original version. You should upload this as a separate file labeled 'Revised Manuscript with Track Changes'.
- An unmarked version of your revised paper without tracked changes. You should upload this as a separate file labeled 'Manuscript'.

If you would like to make changes to your financial disclosure, please include your updated statement in your cover letter. Guidelines for resubmitting your figure files are available below the reviewer comments at the end of this letter.

If applicable, we recommend that you deposit your laboratory protocols in protocols.io to enhance the reproducibility of your results. Protocols.io assigns your protocol its own identifier (DOI) so that it can be cited independently in the future. For instructions see: https://journals.plos.org/plosone/s/submission-guidelines#loc-laboratory-protocols. Additionally, PLOS ONE offers an option for publishing peer-reviewed Lab Protocol articles, which describe protocols hosted on protocols.io. Read more information on sharing protocols at https://plos.org/protocols?utm_medium=editorial-email&utm_source=authorletters&utm_campaign=protocols.

We look forward to receiving your revised manuscript.

Kind regards,

Claudia Isabella Pogoreutz
Academic Editor
PLOS ONE

Reviewer #1: This is a great new methodology which will be of value to the scientific community and presents a novel method of transforming otherwise unsuccessfully transformed non canonical strains. The protocol is reasonably well presented but as it is, it requires a great deal of background understanding due to the large amount of undefined jargon and a lack of description of the context of the experiments performed. As such I believe it should be slightly re-written to make it more accessible, and connect the dots of the rationale.

- General introduction – I highly recommend introducing the rpoN gene in the introduction to give context to the rest of the paper and the hypothesis and proof of principle testing. I.e. motility assay.

Description of rpoN is included as recommended.

- Line 36-39 sentence needs to be re-structured grammatically

Re-structured as requested.

- 55-56 sentence have led to -the development of - molecular tools to enhance..

Inserted as suggested.

Methods

- Maybe good to present evidence to support that their strain FQ-A001 isn't receptive to attTn7 Tn7 insertion, compared to the ES114 WT at this stage of the protocol. In addition, context on why this particular strain was chosen.

This data was included in the original manuscript as Fig. S1. Context as to why FQ-A001 is significant has been added to the introduction.

- T7 integration assay - How did they select against the donor cells harbouring the plasmids which included erm resistance? Was there another selection step to clear the E. coli?

No counterselection was necessary because the higher-salt medium and lower incubation temperature (28°C) promote the growth of *V. fischeri* over *E. coli*. This detail is now stated in the manuscript.

- I suggest you define terms like σ54 and Ptrc, KpnI/SalI and explain their relevance.
These terms have been defined, as suggested.

Genome sequencing and analysis
- Line 119 – Perhaps the FQ-A001 strain with the Tn7::erm marker needs a new name at this point to avoid confusion?
We have removed this phrase to generalize the methodology section. The strain names (AGC020, AGC028, etc.) are listed in Tables 1 & 3.

- Was any genome sequencing or analysis or bioinformatics performed to determine if there were enough homologous regions to facilitate their method before attempting the natural transformation.
The extent of homology required for natural transformation in *V. fischeri* is unknown. However, the progressiveMauve analysis (PMID: 20593022) suggests blocks of homology exist near the Tn7 insertion site, which is captured in the statement and cited figure: "The genomes of ES114 and FQ-A001 exhibit significant homology near the Tn7 insertion site (Fig. 1A)."

- Line 138-139 – This needs to be rephrase for clarity "Crossover sites for homologous recombination events were called as the SNPs corresponding to TIM313 detected in the transformant furthest from the attTn7 site.".
Rephrased as requested.

Motility assay
- This requires context. As it is, the reader may find it difficult to understand the purpose of this assay. Please expand on why this assay was performed and how it pertains to confirming the transformation was successful.
Introduction now provides requested context for motility.

Expected results (why not just, results?)
I believe that the results need to be re-formatted and re-written to be more understandable and clear with the outcomes of this study. Perhaps adding subheadings would be helpful? I think adding part of Figure S2 would help the reader understand the context of the motility assay i.e. S2.D.
Expected results are a format requirement for PLoS ONE Protocols. However, we have included subheadings to increase clarity, as suggested. Because the suggested change to include the background and significance of *rpoN* in the introduction section has improved the manuscript, we have chosen to keep the Figure S2 as is.

191-192 – Should this sentence go at the end of the document?
Moved to the end of the paragraph, as suggested.

Table 3 –
- We need context for what SJSX01000002 is here.
Footnote with context has been added to Table 3, as requested.
